# Cardiac-specific troponin-I (cTnI) in a post-mortem setting

**DOI:** 10.1007/s00414-025-03526-x

**Published:** 2025-05-23

**Authors:** Ethan D. Sutton, Sarah Parsons, Maria Pricone, Hans H. de Boer

**Affiliations:** 1 Bachelor of Biomedical Science Honours, School of Public Health and Preventive Medicine, Monash University, 553 St Kilda Road, Melbourne, VIC 3004 Australia; 2 Dept. of Pathology, Victorian Institute of Forensic Medicine, 65 Kavanagh Street, Southbank, VIC 3006 Australia; 3 Dept. of Toxicology, Victorian Institute of Forensic Medicine, 65 Kavanagh Street, Southbank, VIC 3006 Australia; 4 Dept. of Forensic Medicine, Monash University, 65 Kavanagh Street, Southbank, VIC 3006 Australia

**Keywords:** Troponin, cTnI, Autopsy, Pathology, Sudden cardiac death, Biochemistry, Blood analysis, Decomposition, Forensic medicine

## Abstract

Cardiac-specific troponin (cTn) is widely used in clinical medicine to support a diagnosis of acute myocardial infarction. Several studies have explored the value of cTn testing in deceased individuals. These studies suggest that -although there are important limitations associated with its use- post-mortem cTn can be useful in selected cases. A decision for post-mortem cTn testing should however be influenced by factors that have not been explored in much detail. This includes the success rate of post-mortem cTn testing, and whether cTn levels are stable after death.

Therefore, this study addresses the post-mortem availability and stability of cardiac-specific Troponin I (cTnI). Post-mortem availability was determined by analysing the success rate in 250 high-sensitivity (hs-)cTnI tests on post-mortem blood samples, and its relationship with variables such as sample location, sample type, post-mortem interval, and decomposition. Post-mortem stability was explored by comparing post-mortem cTnI levels between two samples from the same individual, taken at different times.

Post-mortem hs-cTnI tests were successful in 86.4% of cases (216/250), with little effect of sex, age, or cardiopulmonary resuscitation. Visible decomposition precluded a successful test. Other variables associated with decomposition (such as increased post-mortem interval) also affected test success negatively. Our results furthermore suggest that cTnI is very unstable post-mortem, with marked differences in hs-cTnI test results between samples from the same individual. The differences were large (on average 18734 ng/L) and not unidirectional. Instability appeared to increase with larger time intervals, but the results were overall erratic and difficult to interpret.

We conclude that hs-cTnI testing results are generally available in a post-mortem setting, but that testing should be performed on the earliest available blood sample. Samples from decomposed individuals should not be tested. Furthermore, the severe instability of cTnI indicates that any post-mortem hs-cTnI result must be interpreted with caution.

## Introduction

Cardiovascular disease remains the global number one cause of death [1, 2], with approximately 20.5 million deaths worldwide in 2021 [3]. A substantial portion of these deaths are due to sudden cardiac death (SCD), defined by the European Society of Cardiology as a ‘*sudden natural death presumed to be of cardiac cause that occurs within 1 h of onset of symptoms in witnessed cases*,* and within 24 h of last being seen alive when it is unwitnessed’* [4]. The most common cause of SCD is ischaemic heart disease (IHD) due to atherosclerotic coronary artery disease [5].

Ascribing a death to IHD can be challenging, especially in absence of a medical history or ante mortem diagnostic information. Many clinical presentations of IHD are non-specific, and a post-mortem external examination is unable to determine an acute underlying cardiac condition with any degree of certainty. At autopsy, only a subset of cases presents with findings considered unequivocal evidence for fatal IHD. Such findings include for instance thrombotic occlusion of a coronary artery (type 1 myocardial infarction) or histological evidence of established acute myocardial infarction [6, 7]. Other deaths attributed to IHD present with chronic changes, such as severe chronic occlusive coronary atherosclerosis or scarring of the myocardium due to previous infarction. The presumed mechanism of death in these cases is cardiac arrythmia, either due to an acute mismatch between myocardial oxygen demand and supply (type 2 myocardial infarction), or due to the arrhythmogenic properties of myocardial fibrosis. Arrhythmias cannot be diagnosed post-mortem. Also, infarction of the myocardium can only be diagnosed macroscopically after approximately 12–24 h, and with standard histological examination after approximately 12–24 h of survival [7]. As a result, attributing a death to IHD is often inherently uncertain and requires a full autopsy to exclude other causes of death.

To increase their diagnostic certainty, practitioners have turned their attention to other markers for sudden cardiac death in general, and acute myocardial infarction specifically. In clinical practice, high-sensitivity (hs) assays for blood levels of cardiac troponin I (cTnI) and cardiac troponin T (cTnT) are often used. In that context, an elevated level of cTnI or cTnT, when combined with the appropriate clinical symptoms and other diagnostic tests, is considered highly diagnostic of acute myocardial infarction [8].

Multiple studies have explored the application of high-sensitivity cTnI and cTnT testing in deceased individuals, suggesting that elevated troponin levels correlate with myocardial damage, and therefore may provide valuable information for death investigations (e.g [9–11]). However, the interpretation of post-mortem troponin levels is challenging [12]. A major shortcoming is that a rise in cardiac-specific troponin (cTn) is a marker for myocardial injury, not for myocardial infarction. Also, cTn may not be yet elevated when death occurred suddenly, whilst in cases with a protracted dying pathway the levels may have increased due to secondary myocardial injury and are therefore not indicative of a primary cardiac cause of death. Furthermore, a diagnostic cut-off value has not been agreed on [12]. These limitations mean that the potential additional value of post-mortem hs-cTn testing must be assessed on a case-by-case basis.

A decision for post-mortem hs-cTn testing is furthermore influenced by various factors that have not been studied satisfactorily yet. For instance, some post-mortem hs-cTn tests are unsuccessful (i.e., do not produce a result) and there is very limited knowledge on whether levels of cTn are stable after death [12–14].

The research in this paper aims to help address these issues. It assesses the effect of various variables on post-mortem availability of cTnI, defined as the rate of a successful hs-cTnI test outcome, and determines the post-mortem stability of cTnI, defined as the change in serum concentration over time. These results can help practitioners to determine whether ancillary testing for cTnI is feasible and whether the obtained result is reliable.

## Materials and methods

This research was performed at the Victorian Institute of Forensic Medicine (VIFM); a statutory authority tasked with the medical investigation of all deaths reported to the Victorian Coroner, in accordance with the Victorian Coroners Act (2008) [15]. Approximately 7500 deaths are referred to VIFM per year, comprising a large variety of unexpected, natural and unnatural deaths.

All study data was retrieved from the case management system of the VIFM (iCMS). This system holds all information related to a case, including all circumstantial information (e.g. police reports, scene photos, and medical documents), the findings of the post-mortem examination (e.g., autopsy, toxicology, and biochemistry results) and any case correspondence.

Post-mortem troponin testing for VIFM cases is performed on serum centrifuged from post-mortem blood samples. To limit post-mortem alterations, the blood sample is centrifuged at our institute as soon as reasonably possible, and the serum is immediately stored at -20 °C. Testing for cTnI is outsourced to the Royal Melbourne Hospital. For this, the serum sample is defrosted, a small amount is transferred to a smaller tube, refrozen and dispatched. The test is performed on the day samples are received on an ARCHITECT STAT High-Sensitivity Troponin-I assay (Abbott Ireland Diagnostic Division, Longford, Ireland). This is a chemiluminescent microparticle immunoassay using an Abbott Architect i2000 analyser [16]. The assay determines the quantity of cTnI in human plasma or serum in nanograms per litre (ng/L).

### Part A: Post-mortem availability of cTnI

The availability of post-mortem cTnI was studied by reviewing the 250 most recent VIFM cases in which hs-cTnI testing was requested. To be eligible for inclusion, the hs-cTnI test had to be requested on (serum from) a human post-mortem blood sample obtained at VIFM. The sample could have been taken from any anatomical location, and be taken at either admission or autopsy, regardless of the cause of death. If two troponin tests were requested on one decedent, the first successful test was included in the study. This reflects the standard VIFM practice of re-testing when no result is obtained after the first attempt. If two tests were performed but both failed, the first failed test was included in the study.

Cases were included retrospectively from the 5th of February 2023. The 250 included cases were autopsied between 28 March 2019 and 8 March 2023. There was no material change in the autopsy and laboratory protocols in this period.

For each included hs-cTnI test, the following variables were recorded:

- The outcome of the test (success or fail) and if the test was unsuccessful, the reason for failure.
- The anatomical location of sample collection, e.g., femoral vein, subclavian vein, or heart.
- The moment of sample collection, i.e., at admission at VIFM or at autopsy.
- Time between death and blood sample collection, given in 12-hour intervals.
- Whether cardiopulmonary resuscitation (CPR) was attempted.
- The deceased’s sex, age, and BMI (in kg/m^2^ and as WHO class [17]).

Additionally, each decedent’s level of decomposition was visually assessed and scored using the Total Decomposition Score (TDS) according to Megyesi et al. [18]. This method assigns quantitative scores to the degree of decomposition of the head and neck, limbs, and torso, which are combined to obtain the TDS. TDS values can range between 3 (no decomposition) and 35 (total skeletonization). Scoring for most cases was performed by one author (EDS). All cases with a score ≠3 or when scoring was ambiguous were reviewed by the research group for final scoring.

The percentage of successful tests was calculated for the entire study cohort and for all categorical variables.

### Part B: post-mortem stability of cTnI

The stability of post-mortem cTnI was investigated by comparing serum cTnI concentrations (in ng/L) between two sets of post-mortem blood samples: one collected upon admission to VIFM and the other at autopsy, by definition sometime later. The study cohort consisted of 42 decedents.

To ensure a diverse range of cTnI levels in the admission samples, the study population consisted of 23 decedents with hemopericardium and 19 without. Hemopericardium was identified by full-body post-mortem CT scan which is routinely performed upon admission to VIFM. Subsequent autopsy data showed that each hemopericardium resulted from either aortic dissection (*n* = 11) or a ruptured ventricle secondary to myocardial infarction (*n* = 12). The former typically causes rapid death without myocardial damage and was therefore expected to have a relatively low cTnI level. The latter indicates extensive myocardial necrosis and was therefore expected to result in higher cTnI levels. The 19 decedents without hemopericardium were all sudden, natural, non-traumatic deaths.

For each case, cTnI was determined twice: once in a post-mortem femoral vein blood sample obtained at admission to VIFM, and once in a femoral vein blood sample collected at autopsy. If possible, sampling was performed contralaterally to any present medical intervention. Cases were excluded if at least one troponin test failed or if there was visible decomposition (TDS > 3).

A total of 19 cases were included prospectively from the project start date (13th of April 2023). 23 cases were included retrospectively. These retrospective cases were admitted to our Institute in the previous 5 months (between 25 November 2022 and 13 April 2023).

For each case, the following variables were recorded:

- The level of cTnI in the admission blood sample, in ng/L.
- The level of cTnI in the autopsy blood sample, in ng/L.
- The time between admission and autopsy, in 3-hour intervals.
- The deceased’s sex, age, and BMI (in kg/m^2^ and as WHO class [17]).
- Whether a test was included retrospectively.

Time between admission and autopsy was recorded in 3-hour intervals to account for small inaccuracies in time recordings. The time between admission and autopsy was not controlled due to operational demands, and the cohort therefore reflected normal operation times at our Institute.

The absolute and relative difference in cTnI level between admission and autopsy samples was determined for each case in the cohort. A Wilcoxon signed-rank test was performed to determine whether any difference between the two samples was statistically significant. The effect of time between samples was assessed by ordering the cases from shortest to longest admission-autopsy interval and visually assess any trends in cTnI differences. Sub analyses were performed to assess the effect of sex, age, BMI, and retrospective inclusion.

All statistical analyses were performed with Statistical Package for Social Sciences (SPSS, version 29).

## Results

### Part A: availability of post-mortem cTnI

Of the 250 included cases, 216 hs-troponin tests returned a successful result (86.4%; 95% CI = 82.2–90.6%). Of the 34 failed tests, 21 were due to a haemolysed sample, 11 due to a lipaemic sample, and 2 due to an insufficient amount of available serum. Of the latter two cases, one showed mild decomposition, and one showed no decomposition (TDS scores of 4 and 3, respectively).

As show in Table 1, age, sex and BMI did not appear to substantially affect test success rate, with success rates ≥ 75% for most categories. Lower success rates were noted for individuals in the 4th decade of their life and individuals in the underweight BMI category. The low number of cases in these categories was however noted. Lower case numbers were also noted for younger or elderly individuals (younger than 29 or older than 80 years) and morbid obese individuals (BMI > 40 kg/m^2^).

**Table 1:** Availability of post-mortem cTnI in relation to sex, age, and body mass index

		Successful test	Unsuccessful test	Total
**Sex**		***N*** **(%)**	***N*** **(%)**	***N***
	*Male*	147 (90.2)	16 (9.8)	163
	*Female*	69 (79.3)	18 (20.7)	87
***Age (years)***	*Range*	17–93	33–90	250
	*Median [Q1 - Q3]*	67 [58–76]	69.5 [58–77]	250
		***N (%)***	***N (%)***	***N***
	*10–19*	1 (100.0)	0 (0.0)	1
	*20–29*	2 (100.0)	0 (0.0)	2
	*30–39*	5 (71.4)	2 (28.6)	7
	*40–49*	23 (92.0)	2 (8.0)	25
	*50–59*	30 (85.7)	5 (14.3)	35
	*60–69*	61 (88.4)	8 (11.6)	69
	*70–79*	64 (82.1)	14 (17.9)	78
	*80–89*	27 (93.1)	2 (6.9)	29
	*90–99*	3 (75.0)	1 (25.0)	4
***BMI (kg/m*** ^***2***^ ***)***	*Range*	16.6–63.6	17.3–47.5	250
	*Median [Q1 - Q3]*	28.85 [25.5–33.0]	30.15 [25.3–36.2]	250
		***N (%)***	***N (%)***	***N***
	*Underweight (< 18.5)*	3 (50.0)	3 (50.0)	6
	*Normal (18.5–24.9)*	44 (89.8)	5 (10.2)	49
	*Overweight (25-29.9)*	78 (89.7)	9 (10.3)	87
	*WHO Class I (30-34.9)*	52 (89.7)	6 (10.3)	58
	*WHO Class II (35-39.9)*	22 (75.9)	7 (24.1)	29
	*WHO Class III (> 40)*	17 (81.0)	4 (19.0)	21

Abbreviations: cTnI: cardiac-specific Troponin-I; BMI: Body mass index

Success rates for the other variables are presented in Table 2. There were slight differences in success rate in cases with or without CPR attempts (91.9% and 78.4%, respectively). The difference in success rate between samples taken at admission to VIFM and samples taken at autopsy was negligible, being less than 2%.

**Table 2:** Availability of post-mortem cTnI in relation to CPR, sample type and location, decomposition, and post-mortem interval

	Successful test	Unsuccessful test	Total
***CPR***	**N (%)**	**N (%)**	**N**
*Yes*	136 (91.9)	12 (8.1)	148
*No*	80 (78.4)	22 (21.6)	102
***Sample type***	**N (%)**	**N (%)**	**N**
*Taken at admission*	180 (86.1)	29 (13.9)	209
*Taken at autopsy*	36 (87.8)	5 (12.2)	41
***Sample location***	**N (%)**	**N (%)**	**N**
*Femoral vein*	215 (87.4)	31 (12.6)	246
*Heart*	1 (50.0)	1 (50.0)	2
*Subclavian vein*	0 (0.0)	1 (100.0)	1
*Thoracic cavity*	0 (0.0)	1 (100.0)	1
***Total Body Score*** ^***1***^	**N (%)**	**N (%)**	**N**
*3*	216 (88.2)	29 (11.8)	245
*4*	0 (0.0)	2 (100.0)	2
*7*	0 (0.0)	1 (100.0)	1
*10*	0 (0.0)	1 (100.0)	1
*15*	0 (0.0)	1 (100.0)	1
***PMI (hours)***	**N (%)**	**N (%)**	**N**
*< 12*	128 (92.8)	10 (7.2)	138
*> 12-<24*	39 (88.6)	5 (11.4)	44
*> 24-<36*	7 (77.8)	2 (22.2)	9
*> 36-<48*	7 (63.6)	4 (36.4)	11
*> 48-<60*	1 (50.0)	1 (50.0)	2
*> 60-<72*	10 (83.3)	2 (16.7)	12
*> 72* ^*2*^	24 (70.6)	10 (29.4)	34

Abbreviations: cTnI: cardiac-specific Troponin-I; CPR: cardiopulmonary resuscitation; PMI: post-mortem interval

^1^ Total Body Score according to Megyesi et al

^2^ Tests collected at PMI > 72 h were collated

All but four blood samples were taken from the femoral vein, which is the standard location of sampling at VIFM. Two were taken from the heart, one from the subclavian artery and one from the thoracic cavity. The sample from the heart returned a successful test result, the other three samples were unsuccessful.

Individuals with no visible decomposition (i.e., a TDS of 3) had a success rate of 88.2%. Any form of visible decomposition (i.e., TDS > 3) was associated with an unsuccessful test.

Analysis of the post-mortem interval (PMI) of the samples demonstrated a decreasing success rate with increasing post-mortem interval. Samples taken in the first 12 h after death had a success rate of 92.8%. The success rate for samples taken between 12 and 24 h after death was 88.6%. The success rate of samples with a longer post-mortem interval ranged between 50 and 83.3%.

### Part B: Stability of post-mortem cTnI

The average time between sampling at admission and at autopsy was between 81 and 84 h (approximately 3.5 days), with a range between 12 and 15 h and 186–189 h. There was a good spread in the data, with one or more data points for almost each 3-hour time interval between 12 and 189 h.

**Fig. 1:**
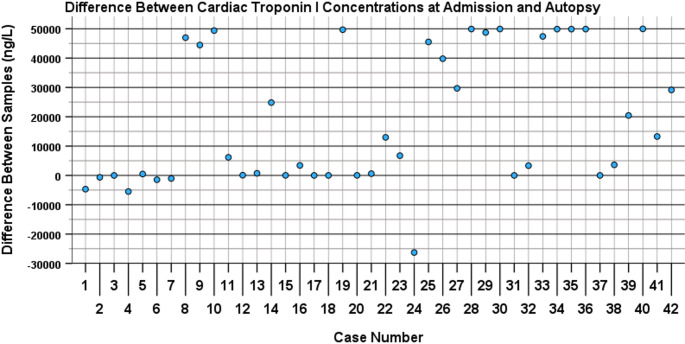
Differences in cardiac troponin I (cTnI, in ng/L) concentration between blood samples collected at admission and autopsy. The cases are ordered on the X-axis based on time interval between admission and autopsy (see Table 1for time intervals). Only a minority of cases show a difference of 0. Differences appear to increase with increased time interval, particularly from case 21 onwards. However, large differences are already observed with shorter intervals whilst later cases returned differences of (close to) 0

The difference between post-mortem cTnI concentrations in admission and autopsy samples was substantial, with an average difference of 18,743 ng/L and a range between − 26,280 ng/L and 49,968 ng/L. The relative difference was equally substantial, with an average difference of 18,725%, with a range between − 53% and 247,486%. Most cases showed an increase of cTnI, but seven of the 42 cases showed a decrease in cTnI concentration between admission and autopsy samples. The results are presented in Table 3 and Fig. 1.

**Table 3:** Stability of post-mortem cTnI concentrations in relation to sampling time interval and whether the case was included retrospectively

	Time between admission and autopsy	cTnI concentrations				Retrospective case
		Admission	Autopsy	Difference		
Case	(hours)	(ng/L)	(ng/L)	(ng/L)	(%)	(yes/no)
1	*> 12-<15*	14,991	10,304	-4687	-31	no
2	*> 15-<18*	4115	3468	-647	-16	no
3	*> 21-<24*	20	10	-10	-50	no
4	*> 21-<24*	28,362	22,861	-5501	-19	no
5	*> 24-<27*	2169	2644	475	22	no
6	*> 33-<36*	4202	2735	-1467	-35	no
7	*> 33-<36*	5952	4896	-1056	-18	no
8	*> 33-<36*	3028	50,000	46,972	1551	yes
9	*> 39-<42*	5544	50,000	44,456	802	yes
10	*> 39-<42*	610	50,000	49,390	8097	yes
11	*> 45-<48*	87	6257	6170	7092	yes
12	*> 48-<51*	258	325	67	26	yes
13	*> 48-<51*	6	726	720	12,000	yes
14	*> 54-<57*	25,165	50,000	24,835	99	yes
15	*> 57-<60*	205	223	18	9	yes
16	*> 60-<63*	1225	4634	3409	278	yes
17	*> 66-<69*	50,000	50,000	0	0	yes
18	*> 69-<72*	50,000	50,000	0	0	yes
19	*> 72-<75*	313	50,000	49,687	15,874	yes
20	*> 78-<81*	7	29	22	314	yes
21	*> 84-<87*	453	1050	597	132	yes
22	*> 84-<87*	2270	15,238	12,968	571	yes
23	*> 87-<90*	43,260	50,000	6740	16	yes
24	*> 87-<90*	50,000	23,720	-26,280	-53	no
25	*> 90-<93*	4484	50,000	45,516	1015	no
26	*> 93-<96*	50	39,848	39,798	79,596	no
27	*> 96-<99*	12	29,704	29,692	247,433	no
28	*> 96-<99*	91	50,000	49,909	54,845	no
29	*> 99-<102*	1274	50,000	48,726	3825	no
30	*> 102-<105*	80	50,000	49,920	62,400	no
31	*> 105-<108*	50,000	50,000	0	0	no
32	*> 108-<111*	11,459	14,790	3331	29	no
33	*> 114-<117*	2599	50,000	47,401	1824	no
34	*> 114-<117*	117	50,000	49,883	42,635	no
35	*> 120-<123*	129	50,000	49,871	38,660	no
36	*> 120-<123*	111	50,000	49,889	44,945	yes
37	*> 138-<141*	303	303	0	0	yes
38	*> 141-<144*	446	4051	3605	808	yes
39	*> 144-<147*	1769	22,206	20,437	1155	yes
40	*> 150-<153*	32	50,000	49,968	156,150	yes
41	*> 177-<180*	304	13,564	13,260	4362	yes
42	*> 186-<189*	20,858	50,000	29,142	140	yes

Abbreviations: cTnI: cardiac-specific Troponin-I; PMI: post-mortem interval

A Wilcoxon Signed-Ranks test confirmed that post-mortem cTnI concentrations in the autopsy samples were statistically significantly different from concentrations in the admission samples. (Z = 694, *p* < 0.0001). Three cases had to be excluded from the test as their admission and autopsy cTnI concentrations were the same (the maximum level of > 50000 ng/L).

In Table 3 and Fig. 1, cases were ordered from shortest to longest time interval between admission and autopsy. This allowed for a visual assessment of whether there is a correlation between lapsed time and cTnI in- or decrease. Especially when the time between admission and autopsy exceeds 84 h (from case 21 onwards in Fig. 1) the difference in cTnI concentration appears to increase markedly. However, the interpretation was severely hampered by a notable number of outliers. Case 17, 18, and 31 had the maximum result of 50,000 ng/L recorded for both samples. If there was a difference in cTnI level between these samples, it could not be elicited from the data.

Table 4 provides the absolute and relative differences in cTnI concentration stratified for sex, age, and BMI. The large differences in cTnI concentration between the autopsy and admission samples were present across the board, without a noticeable trend or correlation with these variables. The analysis was limited by a low number of cases for certain categories, especially for age and BMI.

**Table 4:** Difference in post-mortem cTnI concentrations between admission and autopsy samples; relation with sex, age, body mass index, and whether the case was retrospectively included

		*N*	Difference in ng/L (mean, min-max)	Difference in % (mean, min-max)
**Sex**				
*Male*		23	22,805 (-26280–49968)	24,726 (-53–247433)
*Female*		19	13,827 (-5501–49920)	11,462 (-35–62400)
***Age (years)***				
*10–29*		2	19,899 (0–39798)	39,799 (1–79596)
*30–39*		3	33,858 (6170–49889)	17,684 (1015–44945)
*40–49*		4	36,895 (720–49968)	58,025 (1551–156150)
*50–59*		4	9946 (-26280–49687)	4168 (-53–15874)
*60–69*		14	19,094 (-1467–49871)	21,663 (-35–247433)
*70–79*		10	14,609 (-5501–49909)	5915 (-50–54845)
*80–89*		5	9018 (-4687–49883)	8527 (-31–42635)
***BMI (kg/m*** ^***2***^ ***)***				
*Underweight (< 18.5)*		2	-2349 (-4687– -10)	-41 (-50– -31)
*Normal (18.5–24.9)*		6	14,515 (0–45516)	964 (1–4362)
*Overweight (25-29.9)*		14	19,401 (-26280–49883)	28,452 (-53–247433)
*WHO Class I (30-34.9)*		13	32,028 (-5501–49968)	28,836 (-19–156150)
*WHO Class II (35-39.9)*		5	2138 (0–6740)	98 (0–314)
*WHO Class III (> 40)*		2	3085 (0–6170)	3546 (0–7092)
***Retrospective case***				
*Yes*		23	16,068 (-5501–49968)	7807 (-50–156150)
*No*		19	21,982 (-26280–49920)	31,944 (-53–247433)

Abbreviations: BMI: body mass index

Special consideration was given to the use of retrospective samples, to see if a longer storage time at -20 degrees Celsius affected the stability of cTnI. Twenty-three cases were included retrospectively, their case numbers are specified in Table 3. In all these cases, a hs-cTnI test had already been performed on the admission sample and the second test was requested on a sample obtained at autopsy. The results of this sub analysis were not straightforward. The mean (ng/L) and relative (%) difference between admission and autopsy samples were large, irrespective of the use of a retrospective sample (see Table 4). A more granular analysis was hampered by an unexpected and very unfortunate clustering of the cases in terms of the time between admission and autopsy. Cases 8–23 and cases 36–42 were included retrospectively, the others did not. As a result, there were only limited numbers of retrospective and prospective cases with a similar interval between admission and autopsy sample. This precluded any meaningful further analysis.

## Discussion

Implementation of any ancillary test requires basic knowledge on whether the test is likely to yield a (reliable) result. This is especially the case when such a test comes at additional labour and monetary costs, as is the case with high-sensitivity cTn testing.

Although many studies have focused on the diagnostic value of cTn in a post-mortem setting, very little attention has been given to the variables that affect test success. For instance, the otherwise comprehensive literature review by Barberi and Van den Hondel [12] does not discuss it. Test success is briefly mentioned in the largest study up to date from Chen et al. [19], stating that haemolysis was an issue in some cases, but without providing further detail. In their 2019 review, Cao et al. suggest that frozen samples may be more affected by haemolysis and therefore should be avoided, but also without providing more data [20].

As such, our study appears to be the first which specifically focuses on variables that may influence the success of post-mortem hs-cTn testing. Our results indicate that hs-cTnI tests performed on post-mortem serum samples were overall successful and that test success is largely independent of demographic variables. A test result success of more than 75% was found in the total cohort and across the decedent demographic categories of age, sex, and BMI, in all groups with 10 or more cases.

As a rule, increased decomposition increases the probability of haemolytic or lipaemic blood samples, which both interfere with hs-cTn testing [19–21]. Our results confirm this, with 32 of the 34 unsuccessful tests due to degradation of the sample. Furthermore, all tests performed on decedents with visible signs of decomposition failed, although this sample was small (*n* = 5). We therefore advise that hs-cTnI tests should not be performed in a context of visible decomposition, although the absence of visible decomposition does not guarantee a successful test.

Lapsed time is one of the most important determinants of decomposition and our results reflect this, with an association between test success and the time between death and sample collection. Overall, success rate diminished with time, especially after a post-mortem interval of 24 h. The trajectory by which success rate diminishes is somewhat erratic, which may be explained by differences in other important determinants of decomposition, such as body temperature at time of death, circumstances at the death scene, and cause of death. The retrospective nature of our study precluded a more detailed analysis of such variables.

It was noted that test success did not differ substantially between admission and autopsy samples, even though autopsy samples are by definition taken at a later time than admission samples. This may be explained by the relatively quick turn-around-time in our institute, coupled with refrigeration of the deceased after admission. These measures are specifically taken to minimize decomposition between admission and autopsy.

The effect of time and/or decomposition on test success was also relevant to interpret the effect of sample location and CPR on test success rate. The femoral vein is the preferred location for a blood sample at VIFM, and other locations are only considered when multiple attempts for a femoral sample are unsuccessful. Often, such cases show visible decomposition, and this can explain the high level of unsuccessful tests in the low number of non-femoral samples. CPR is usually only performed in individuals with a witnessed collapse or who were last seen alive a short time before their death. By extension, the post-mortem interval and the level of decomposition can be assumed to be lower in the cohort that was resuscitated than in the cohort without resuscitation. This may explain the lower test success rate in the non-CPR cohort.

Our study demonstrated surprisingly different cTnI levels when comparing admission samples with autopsy samples from the same individual, suggesting that cTnI is very unstable in blood post-mortem. This instability did not appear to correlate with sex, age and BMI categories, in line with earlier publications [e.g., 20]. The results were erratic and difficult to interpret but overall, instability did appear to be time dependent.

The reasons for the demonstrated differences are not entirely clear. One of the proposed mechanisms is autolysis, which might cause leakage of troponin from cardiomyocytes into the blood and other tissues [22]. This would explain the increase of cTnI over time, but it cannot account for the decrease which was observed in seven out of 42 cases. Notably, almost all of the cases in which cTnI level decreased did so after a relatively short interval between admission and autopsy, i.e. less than 36 h.

Comparison of our results with previous work is challenging since the literature on post-mortem stability of cTnI or cTnT is conflicting. This was already noted by Palmiere et al. in 2018 [22],, who summarized the literature on the subject, and stated that Zhu et al. [14, 23, 24], Remmer et al. [25], and Chen et al. [19] found time-dependent differences in post-mortem troponin levels, while Pérez-Carceles et al. [26] and Peter et al. [27] did not. The study of Gonzales-Herrera [28] also found a time-dependent increase. These studies all have methodological limitations though. They did not, like the current study, test two samples from the same person at different points in time. Rather, they inferred stability from comparing results between selectively included cases which were stratified according to post-mortem interval. Our methodology and results reflect that of Moridi et al. [13] who compared high-sensitivity cTnT test results at admission and autopsy and found significant differences between them. Combining our results with the existing literature, we conclude that there is considerable evidence to suggest that cTnI and cTnT are markedly unstable in post-mortem blood under normal operational conditions at our Institute, especially with increased PMI.

Whether this instability is relevant depends on how a hs-cTn result is interpreted. It is generally agreed upon that ‘normal’ post-mortem levels of cTnI are much higher than ante mortem levels, and that the clinical cut-off value (the 99th percentile) of 40 ng/L [29, 30] has no relevance in a post-mortem setting. An alternative cut-off value has not been agreed on. Such a cut-off value is likely to depend on the specific diagnostic issue and circumstances of the case.

It has been suggested that frozen blood samples should be avoided for biochemical analysis (Cao et al., Barberi et al.) due to the likelihood of haemolysis. Our institute uses frozen serum samples, not whole blood samples. This should prevent further haemolysis once the serum sample is obtained. Storage of the serum samples at -20 directly after centrifuging, and only defrosting a sample for analysis aims to further limit degradation. The defrosting and refreezing required for transportation could theoretically have affected stability, but our study design was unable to examine this in more detail. Various other studies also used frozen serum samples, apparently without major issues (e.g [9, 10, 31, 32]).

To confirm whether cTnI levels indeed remain stable in serum at -20 degrees Celcius, we attempted to compare the subset of older (retrospective) cases with cases with limited serum storage time (prospective cases). An unfortunate and unexpected clustering of cases in terms of post-mortem interval however precluded this sub analysis. The use of older frozen samples cannot always be avoided, since the need for a hs-cTn test may not be readily apparent at the time when the sample is taken. Further research is therefore needed to determine if storage at -20 degrees Celsius indeed limits further degradation of the sample.

Our study has several limitations. First, it was performed in the context of normal operations at our institute. As a consequence, many variables were deliberately unaccounted for. For instance, we did not selectively include decedents with a specific PMI, and we did not regulate the amount of time between blood samples or laboratory processes in Part B of the study. This hampered interpretation of some of the results. For instance, minor variability in the time between obtaining and centrifuging blood samples could theoretically have confounded our results on cTnI stability. At the same time, conducting our research in the setting of normal operations ensured our results are representative of (and applicable to) our daily practice. The workflow in our institute is likely similar to many large forensic pathology institutes, making the results also transferrable to their situation.

Another limitation related to sample size. Sample sizes were dependent on inherent financial, operational, and time constraints, resulting in low case numbers in some subcategories. For example, the 10–19, 20–29, 30–39, 90–99 age groups and the underweight BMI category were underrepresented in the data. Future research is needed to address this.

Lastly, our only studies focused on cTnI, as cTnT testing is not available at our institute. It is assumed that many of the processes that affect the availability and stability of cTnI are the same for cTnT, but this has not been confirmed experimentally.

## Conclusion

Post-mortem high-sensitivity cTnI tests on serum samples are successful most of the time, but success can be compromised by longer post-mortem intervals and testing is likely to be unsuccessful in case of visible decomposition. In our institute, post-mortem cTnI levels exhibit significant instability, with large differences between cTnI concentrations in admission samples and autopsy samples from the same individual. This instability appears to increase with increased PMI, but outliers are noted, and the instability is not linear or unidirectional. Post-mortem cTnI levels should therefore be interpreted with great caution.

## Data Availability

The data that support the findings of this study are available from the corresponding author upon reasonable request.
